# Butyrate and Metformin Affect Energy Metabolism Independently of the Metabolic Phenotype in the Tumor Therapy Model

**DOI:** 10.3390/biom11121831

**Published:** 2021-12-04

**Authors:** Felix B. Meyer, Christian Marx, Sonja B. Spangel, René Thierbach

**Affiliations:** 1 Human Nutrition Research Group, Institute of Nutritional Sciences, Friedrich Schiller University Jena, 07743 Jena, Germany; felix.meyer@uni-jena.de (F.B.M.); sonja.barbara.spangel@uni-jena.de (S.B.S.); 2 Leibniz Institute on Aging—Fritz Lipmann Institute (FLI), 07745 Jena, Germany; christian.marx@leibniz-fli.de

**Keywords:** cancer metabolism, tumor heterogeneity, monoclonal cell lines, metabolic phenotype, butyrate, metformin, tumor therapy

## Abstract

The BALB/c cell transformation assay (BALB-CTA) considers inter- and intra-tumor heterogeneities and affords the possibility of a direct comparison between untransformed and malignant cells. In the present study, we established monoclonal cell lines that originate from the BALB-CTA and mimic heterogeneous tumor cell populations, in order to investigate phenotype-specific effects of the anti-diabetic drug metformin and the short-chain fatty acid butyrate. Growth inhibitory effects were measured with a ViCell XR cell counter. The BALB/c tumor therapy model (BALB-TTM) was performed, and the extracellular glucose level was measured in the medium supernatant. Using a Seahorse Analyzer, the metabolic phenotypes of four selected clones were characterized, and effects on energy metabolism were investigated. Anti-carcinogenic effects and reduced glucose uptake after butyrate application were observed in the BALB-TTM. Metabolic characterization of the cell clones revealed three different phenotypes. Surprisingly, treatment with metformin or butyrate induced opposite metabolic shifts with similar patterns in all cell clones tested. In conclusion, the BALB-TTM is a relevant model for mechanistic cancer research, and the generation of monoclonal cell lines offers a novel possibility to investigate specific drug effects in a heterogeneous tumor cell population. The results indicate that induced alterations in energy metabolism seem to be independent of the original metabolic phenotype.

## 1. Introduction

About a century ago, Otto Warburg described abnormalities in the energy metabolism of malignant cells reflected by increased aerobic glycolysis and reduced oxidative metabolism [1,2], which is nowadays known as the Warburg effect. In 2011, the complexity of tumor cell metabolism was added as a new Hallmark of Cancer [3], underlining its relevance for cancer research. Nevertheless, the initial generalized assumption of just a switch towards aerobic glycolysis in transformed cells is obsolete nowadays. Rather, cancer cells seem to show both enhanced glycolysis and oxidative metabolism simultaneously [4]. Besides this, several other metabolic changes promote cancer development—so-called oncometabolites. These are categorized as transforming activities that directly contribute to malignant cell transformation, enabling activities that are altered in consequence of cell transformation and neutral activities that are expendable for the tumor [5]. Regarding these facts, it is obvious that targeting cell metabolism plays a crucial role for current cancer treatment approaches [6].

The tumor cell metabolism corresponds to its non-malignant tissue of origin and can change during progression [7,8], explaining the heterogeneity among different types of tumors. Moreover, in response to chemotherapy, cancer cells can rewire their metabolism, which contributes to an adaptive resistance. This heterogeneity challenges the successful treatment of tumor metabolism as an anti-cancer target, although targeting tumor metabolism in combination with standard chemotherapy could help to overcome drug resistance and increase drug efficiency [9,10]. In order to understand pivotal metabolic changes in different cell types, simplified in vitro cell culture models are essential. The BALB/c cell transformation assay (BALB-CTA) mimics different phases of malignant cell transformation in vitro and is well suited for molecular cancer research [11,12]. In contrast to established human cancer cell lines, the alterations in cell metabolism can be traced during the whole process of malignant cell transformation with the BALB-CTA. Based on this method, we recently established the BALB/c tumor therapy model (BALB-TTM), in which transformed cells are treated therapeutically in the late phase of the assay [13].

Different effects on the metabolism of untransformed and malignant cells are described for the short-chain fatty acid (SCFA) butyrate and the anti-diabetic drug metformin. While the histone deacetylase (HDAC) inhibitor butyrate stimulates the proliferation of fetal human colonocytes, it impairs the growth of cancer cells exhibiting the Warburg effect [14]. The electron transport chain (ETC) complex I inhibitor metformin affects the glucose consumption of cells by enhancing glycolysis, for example, in peripheral tissues like muscle and kidney [15,16], but by also reducing glycolysis in cancer cell lines [17,18,19,20,21]. However, the comparability of these studies is limited, because all cell lines used were derived from different tissues with different developmental stages. Using the BALB-TTM, a direct comparison of non-malignant and transformed cells from the same origin is possible and offers new opportunities for the understanding of cancer cell metabolism during its development and treatment.

The aim of the present study was to investigate cell-type-specific effects of butyrate and metformin. A limiting factor of the BALB-TTM is the co-existence of untransformed monolayer cells and malignant transformed cell foci in the same well, making it nearly impossible to observe cell-type-specific effects. Therefore, we generated monoclonal cell lines that were isolated from type-III cell foci of the BALB-CTA in order to enable a direct and more specific comparison between untransformed and malignant cells. Thus, distinct metabolic phenotypes of individual cell clones that were differently affected by butyrate and metformin were characterized.

## 2. Materials and Methods

### 2.1. Cell Culture

Dr. A. Poth (Harlan Cytotest Cell Research GmbH, Roßdorf, Germany) kindly provided us with BALB/c-3T3-A31-1-1 cells from the Hatano Research Institute of Japan that were used for all of the experiments. The cells were cultivated with DMEM/HAM’s F-12 (Biochrom #T481, Berlin, Germany) containing 3.15 g/L D-glucose, 5% fetal bovine serum, and 1% penicillin/streptomycin in an incubator (37 °C, 5% CO_2_, 95% humidity). Only sub-confluent grown cells (70 to 80% confluence) between passages 20 and 45 were used. Tests for mycoplasma infections were conducted regularly and were negative.

### 2.2. Trypan Blue Cell Count

Non-transformed BALB/c cells or the monoclonal cell clones were seeded into 6 cm dishes (50,000 per dish) or 6-well plates (25,000 per well) and treated with different concentrations of butyrate 24 h later. The numbers of living and dead cells were determined with a ViCell XR cell counter (Beckman Coulter, Krefeld, Germany) using trypan blue after 48 h of incubation.

### 2.3. BALB/c Tumor Therapy Model

**Figure 1:**
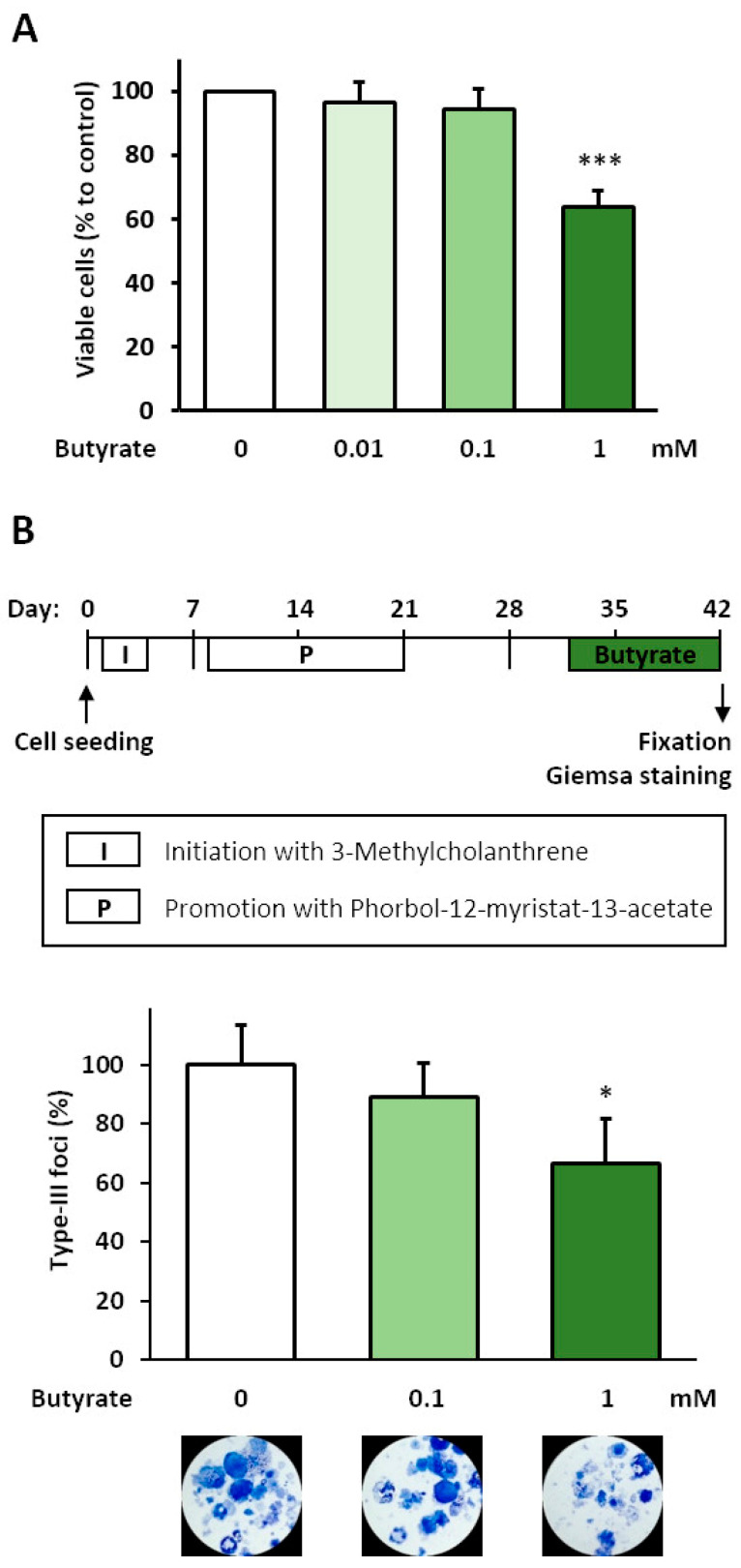
Anti-carcinogenic effect of butyrate in the BALB-TTM. (**A**) Untransformed BALB/c cells at a confluence of 30% were treated with 1 mM butyrate for 48 h. Afterwards, living and dead cells were counted with a ViCell XR cell counter using trypan blue. (**B**) The BALB-TTM was used (see methods). Butyrate was applied in the late phase of malignant cell transformation on Days 32–42. Representative pictures of Giemsa-stained cells and the relative numbers of type-III foci are shown. Statistical differences were calculated from three biological replicates (mean + SD) via a one-way ANOVA (post hoc: two-sided Dunnett-T for the cell count and Bonferroni for the BALB-TTM) with * *p* < 0.05 and *** *p* < 0.001 vs. control.

**Figure 2:**
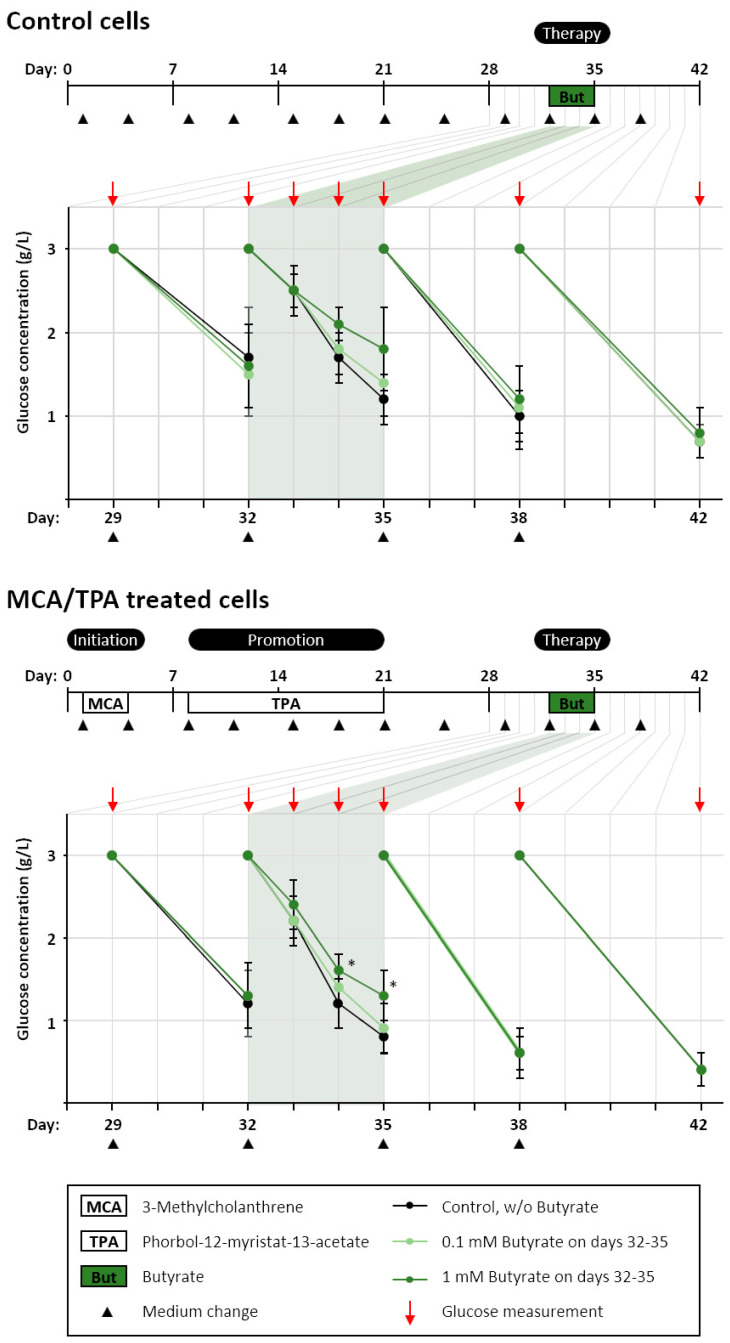
Butyrate alters glucose consumption in the BALB-TTM. The BALB-TTM was performed (see methods). An additional treatment with butyrate was conducted on Days 32–35. On Days 35–42, cells were incubated in medium without butyrate. Control cells were treated with DMSO instead of MCA/TPA. Medium supernatants were collected at Days 32, 33, 34, 35, 38, and 42, and the glucose concentration was measured. The slanting lines indicate a decrease in glucose compared to fresh medium with 3.0 g/L D-glucose, which was replaced every 3–4 days. Statistical differences were calculated from four biological replicates via a one-way ANOVA (post hoc: two-sided Dunnett-T) with * *p* < 0.05 vs. control for each point in time.

The BALB-TTM was performed as described previously [13]. In brief, 5000 cells were seeded into Corning^®^ Primaria™ 6-well plates (Corning #353846, Corning, NY, USA) on Day 0 and cultivated under standard conditions. Malignant cell transformation was induced by treatment with the tumor initiator 3-methylcholanthrene (MCA, Sigma-Aldrich #213942, St. Louis, MO, USA) (0.5 µg/mL dissolved in DMSO) on Days 1–4, followed by the tumor promotor 12-tetradecanoylphorbol-13-acetate (TPA, Sigma-Aldrich #79346, St. Louis, MO, USA) (0.3 µg/mL dissolved in DMSO) on Days 8–21. Consequently, cells lost their contact inhibition and started to grow over the monolayer, and as a result, characteristic cell foci of transformed cells were formed. An additional, therapeutic treatment with 1 mM sodium butyrate (dissolved in water) (Sigma-Aldrich #8.17500, St. Louis, MO, USA) was performed on Days 32–42 (Figure 1B) or on Days 32–35 (Figure 2). On Day 42, cells were fixed with methanol and Giemsa stained for better visualization of the cell foci. For all experiments, DMSO served as a solvent control and was adapted to a constant concentration of 0.05% from Day 1. Unless stated otherwise, the assays were performed with four technical replicates in four biological experiments. The number of type-III foci was counted independently by two different persons as described elsewhere [22].

### 2.4. Glucose Measurement

The glucose concentration was measured colorimetrically in the medium supernatant using a glucose assay kit (Sigma-Aldrich #GAGO20, St. Louis, MO, USA) as described previously [13]. For these experiments, medium without phenol red was used with 3.0 g/L glucose. When measuring glucose during the BALB-TTM at several points in time, normalization to the total cell number was not possible because of the duration of the assay and the ongoing proliferation of transformed cells.

### 2.5. Generation of Monoclonal Cell Lines

A BALB/c cell transformation assay was performed as described elsewhere [11,13] in six Corning^®^ Primaria™ 6-well plates (Corning #353846, Corning, NY, USA). From the total of 36 wells, 6 served as a solvent control that were only treated with DMSO and 30 wells were treated with MCA/TPA in order to induce malignant cell transformation. On Day 35 of the assay, after the formation of characteristic cell foci, one focus of each well was chosen, and cells were isolated with cloning rings [23]. Using sterile forceps, stainless steel cloning rings with a diameter of 8 mm were gently pressed in sterile silicone grease (Sigma-Aldrich #Z273554, St. Louis, MO, USA) and placed over the foci of interest. By gently pressing, a seal between the plate and the ring was created. The encircled cell focus, consisting of transformed cells, was trypsinized, and a 4 µL cell suspension was transferred into the first well of a 96-well plate (A1) with 196 µL of medium. The monolayer of untransformed control cells underwent the same procedure. The cell suspensions from well A1 were diluted gradually. At first, this was performed vertically at 1:2 for each well until well H1. Afterwards, each well of the first column was gradually diluted horizontally 1:2 until column 12. As a result, there was at least one well per 96-well plate where only one single cell was seeded. These wells were spotted after 6–8 days, whereby one single cell focus in the well indicated the monoclonal origin. When these monoclonal cells reached a confluence of 90% (after 18–22 days), they were trypsinized and further cultivated in cell culture dishes. From the total of 36 cell clones, 7 were discarded due to very slow to no cell growth. Untransformed, DMSO-treated cells were declared as wild-type (WT) 41–46 cells, and transformed, MCA/TPA-treated cells were declared as transformed (T) 50–79. Cell cryos were generated of each clone at a confluence of 70%. The original cells were also cryo conserved on Day 0 after the seeding of the BALB-CTA and declared as original cells (OC) 40.

### 2.6. Giemsa Staining of Monoclonal Cell Clones

A total of 25,000 cells of each cell clone were seeded into two wells of a Corning^®^ Primaria™ 6-well plate (Corning #353846, Corning, NY, USA) and cultivated with regular medium changes every 3–4 days. After 16 days, the cells were fixed with methanol and stained with Giemsa solution following the same procedure as that used for the BALB-TTM on Day 42. This phenotypical documentation was performed twice.

### 2.7. Growth Curves

Growth curves were generated for the 30 cell clones using sulforhodamine B (SRB) dye (Sigma-Aldrich #S9012, St. Louis, MO, USA) [24]. For each clone, 25,000 cells were seeded into 6-well plates (TPP #92406, Trasadingen, Switzerland). Fixation of the cells was conducted after 24, 32, 48, 56, 72, 80, 96, and 104 h with 50% trichloroacetic acid (AppliChem #141067, Darmstadt, Germany). Afterwards, cells were stained with SRB (Sigma-Aldrich #S9012, St. Louis, MO, USA) and washed with 1% acetic acid (Carl Roth #3738.4, Karlsruhe, Germany). For the quantification of protein-bound dye, it was solubilized by adding 10 mM tris buffer (AppliChem #A1379, Darmstadt, Germany), and the optical density was measured at 560 nm. The absorption was plotted semilogarithmically against time, and cell duplications per 24 h were calculated within the linear range of the growth curve. This experiment was performed only one time.

### 2.8. Seahorse Analysis of Cells

Distinct cell numbers were seeded into 24-well Agilent Seahorse XF Cell Culture Microplates (Agilent Technologies #102342-100, Waldbronn, Germany) for each cell clone, in order to reach a confluence of 90% for the Seahorse XF Cell Mito Stress assay after 48 h.

**Figure 3:**
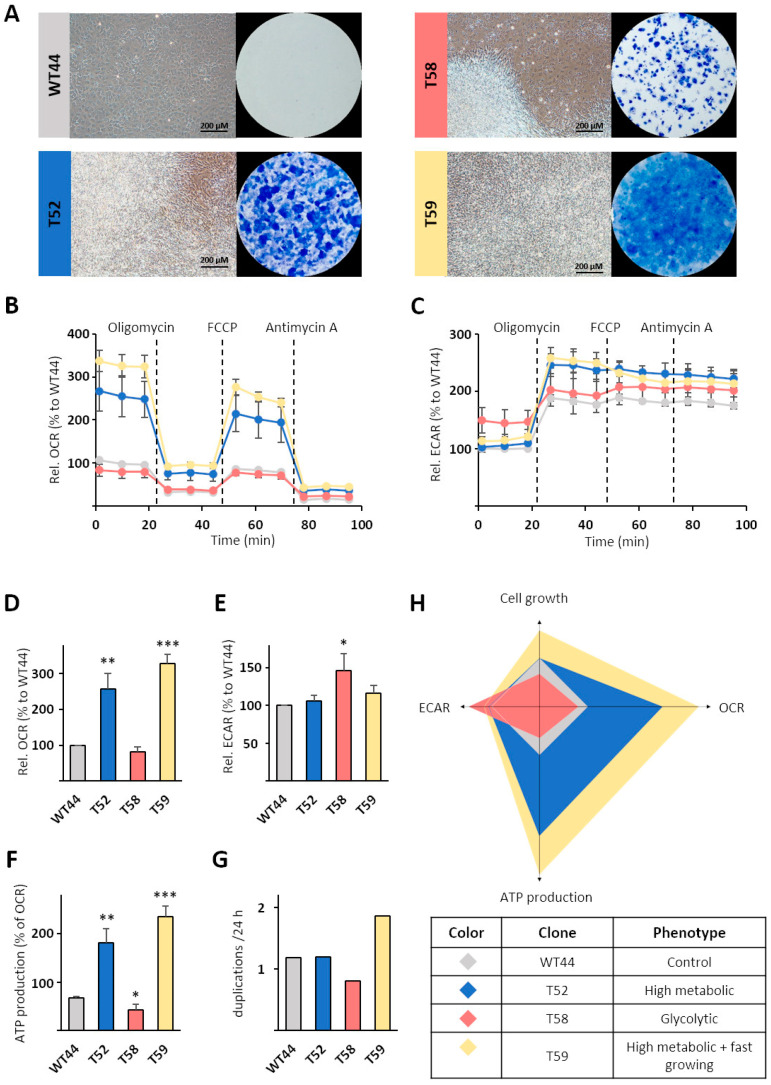
Characterization of monoclonal cell lines. The metabolism of the non-transformed cell clone WT44 and the three MCA/TPA transformed cell clones T52, T58, and T59 was analyzed. (**A**) Cells were cultivated for 16 days, fixed with methanol, and stained with Giemsa solution. A Cell Mito Stress Test was performed using an XFe24 Analyzer and (**B**) the oxygen consumption rates (OCRs) and (**C**) extracellular acidification rates (ECARs) were measured simultaneously. (**D**) Basal respiration and (**F**) mitochondrial ATP production were calculated from OCR values. (**E**) All values are shown relative to the non-transformed cell clone WT44. (**G**) Cell clones were fixed at several points in time and their cell density was quantified with sulforhodamine B (SRB) dye (see methods). The duplications per 24 h were calculated within the linear range of the resulting growth curves from one experiment. (**H**) The different metabolic phenotypes of individual cell clones are illustrated. Data in (**B**–**F**) are shown as the mean + SEM from three biological replicates. Statistical differences were calculated via a two-sided *t*-test with * *p* < 0.05, ** *p* < 0.01, and *** *p* < 0.001 vs. WT44.

Direct comparison between cell clones (Figure 3): Supernatant medium was replaced with Seahorse XF Base Medium (Agilent Technologies #103334-100, Waldbronn, Germany, pH adjusted to 7.4 with NaOH), supplemented with 10 mM D-glucose (Sigma Aldrich #G7021, St. Louis, MO, USA), 2 mM L-glutamine (Thermo Fisher Scientific #25030081, Waltham, Mass, USA), and 1 mM sodium pyruvate (Thermo Fisher Scientific #11360070, Waltham, Mass, USA). Cells were then incubated in a CO_2_-free incubator at 37 °C for an additional hour. With a Seahorse XFe24 Analyzer (Agilent Technologies, Waldbronn, Germany), the basal oxygen consumption rate (OCR) and extracellular acidification rate (ECAR) were measured before adding 2 µM oligomycin (Sigma Aldrich #04876, St. Louis, MO, USA), 2 µM carbonyl cyanide-p-trifluoromethoxyphenylhydrazone (FCCP) (Abcam #ab120081, Cambridge, UK), and 2 µM antimycin A (Sigma Aldrich #A8674, St. Louis, MO, USA). Each compound was injected consecutively. The OCR and ECAR were measured in picomoles per minute and milli-pH units per minute, respectively, in cycles of 3 min mix and 3 min measure periods at 37 °C. At the end of the measurement, cell densities per well were quantified by crystal violet staining as previously described [25] and the observed OCR and ECAR were normalized to the corresponding cell densities. Wave Desktop 2.6 software (Agilent Technologies, Waldbronn, Germany) was used to analyze the datasets.

**Figure 4:**
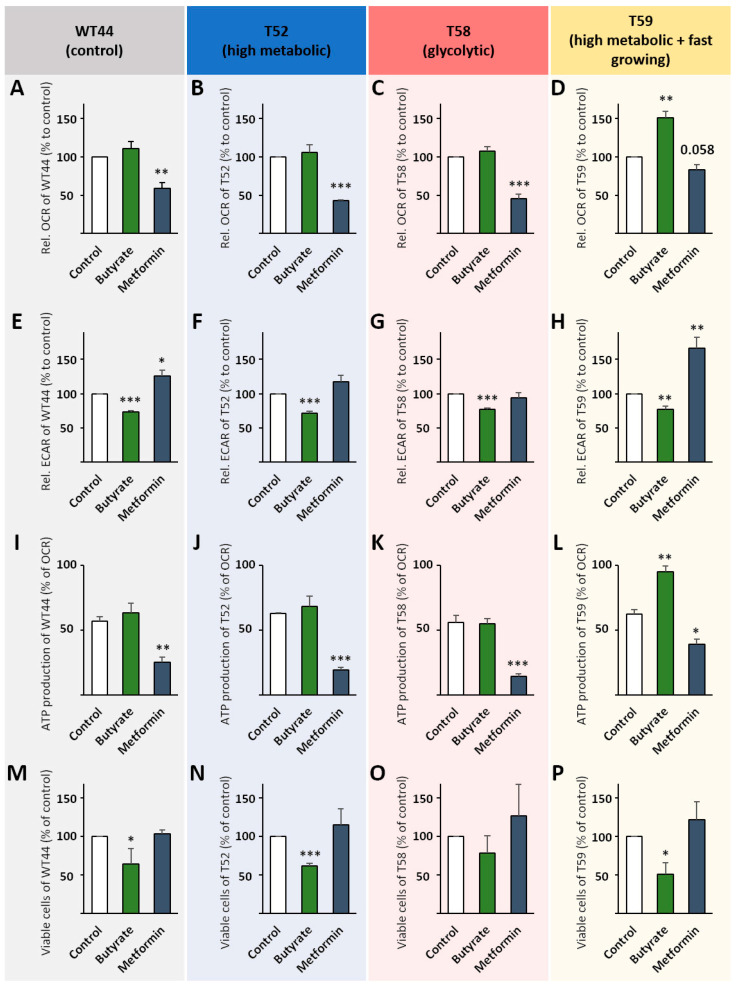
Metabolic changes after metformin and butyrate treatment. Cell clones were seeded in 24-well Agilent Seahorse XF Cell Culture Microplates, then treated with 1 mM butyrate or 1 mM metformin for 24 h followed by a Cell Mito Stress Test using a Seahorse XFe24 Analyzer. Mitochondrial ATP production and basal respiration were calculated from OCRs for WT44 (**A**,**I**), T52 (**B**,**J**), T58 (**C**,**K**), and T59 (**D**,**L**). Basal ECARs are shown for WT44 (**E**), T52 (**F**), T58 (**G**), and T59 (**H**). The number of viable cells was determined after 24 h of treatment with butyrate or metformin with a ViCell XR cell counter using trypan blue for the clones WT44 (**M**), T52 (**N**), T58 (**O**), and T59 (**P**). All values are shown relative to the untreated controls of each cell clone as the mean + SEM for the Seahorse measurements or as the mean + SD for cell numbers from three or four biological replicates. Statistical differences were calculated via a two-sided *t*-test for the Seahorse measurements or a one-way ANOVA following a Bonferroni or Dunnett-T3 post hoc test for the viable cells with * *p* < 0.05, ** *p* < 0.01, and *** *p* < 0.001 vs. untreated control cells.

Butyrate and metformin treatment of individual cell clones (Figure 4): Treatment with 1 mM butyrate or 1 mM metformin was performed 24 h before the measurement. Supernatant medium was replaced with Seahorse XF DMEM Medium pH 7.4 (Agilent Technologies #103575-100, Waldbronn, Germany), supplemented with 10 mM D-glucose, 2 mM L-glutamine, and 1 mM sodium pyruvate. All the following steps were similar to those for the above-mentioned analysis.

### 2.9. Statistical Analysis

The software products IBM SPSS Statistics Premium 28 (Armonk, NY, USA) and Microsoft Excel 2016 (Redmond, WA, USA) were used for all statistical analyses. The results were tested for homogeneity of variances in each case, and as described elsewhere [26,27], the normal distribution was neglected. For the cell viability assay and the glucose measurement, a one-way ANOVA was performed following the Dunnett-T2 post hoc test in case of homogeneity of variances or the Dunnett-T3 if this was not the case. Statistical differences of the type-III foci in the BALB-TTM were calculated for existing homogeneity of variances via a one-way ANOVA and an additional Bonferroni post hoc test. Otherwise, a Dunnett-T3 post hoc test was performed. Statistical differences in the Seahorse measurements were calculated via a two-sided *t*-test.

## 3. Results

### 3.1. Analysis of Anti-Carcinogenic Effects of Butyrate in the BALB-TTM

In order to find the optimal concentrations for therapeutic application, the growth inhibitory and cytotoxic effects of butyrate were tested in untransformed BALB/c cells. After the incubation of proliferating sub-confluent cells with 0.01, 0.1, and 1 mM butyrate for 48 h, the number of viable cells decreased significantly only after exposure to 1 mM butyrate, by 36% (Figure 1A). The number of dead cells, however, remained unchanged after all butyrate treatments, and the cell viability was always >95% (not shown), indicating an anti-proliferative effect. Thus, we used 0.1 and 1 mM butyrate in the BALB-TTM. Compared to DMSO, treatment with 1 mM butyrate for 10 days (Days 32–42) decreased the number of malignant type-III foci significantly by 34%, whereas 0.1 mM showed only moderate effects (Figure 1B).

### 3.2. Glucose Consumption after Butyrate Treatment in the BALB-TTM

In cancer cells, butyrate is known to affect their energy metabolism by reducing their glucose consumption [28,29], which is described as a reversion of the Warburg effect and could contribute to butyrate’s anti-cancer effectiveness. Hence, we evaluated the changes in the glucose concentration in the supernatant cell culture medium before and after butyrate treatments in the BALB-TTM. Control cells were treated with DMSO instead of MCA/TPA during the BALB-TTM and were thus not transformed (Figure 2). In order to prove long-term and adaptive effects, cells and cell foci were treated for only 72 h (Days 32–35) with 0.1 or 1 mM butyrate and were further cultivated until Day 42 without butyrate. Glucose levels were measured before butyrate was added (Day 32) and 24, 48, and 72 h after the addition of butyrate (Days 33, 34, and 35), as well as on Days 38 and 42 in the absence of butyrate. The cell culture medium was refreshed every 3–4 days during the analysis to recover the initial glucose concentrations (3.0 g/L) and, thus, to always have excessive amounts of glucose in the medium. Before butyrate was added, the average glucose consumption was in the same range for each group (Day 32) with remaining concentrations of 1.5–1.7 g/L for control cells and 1.2–1.3 g/L for MCA/TPA-treated malignant cells/cell foci. Treatment with butyrate for 3 days (Day 35) decreased the glucose consumption in both control cells and malignant cells/cell foci in a dose-dependent fashion. In control cells, the medium glucose concentration after 3 days in culture ranged from 1.2 g/L for untreated cells to 1.4 g/L after treatment with 0.1 mM butyrate and 1.8 g/L after treatment with 1 mM butyrate (0.6 g/L difference). In MCA/TPA-treated cells, where a mixed culture of malignant transformed, foci-forming cells and non-transformed monolayer cells existed, 1 mM butyrate significantly decreased the glucose consumption. Compared to untreated cells, significantly higher remaining glucose concentrations were observed on Day 34 (1.2 g/L vs. 1.6 g/L) and Day 35 (0.8 g/L vs. 1.3 g/L) after treatment with butyrate, confirming its effect on tumor cell metabolism. Adaptive effects, when the cells were released from butyrate, were observed neither for control cells nor for MCA/TPA-treated cells on Days 38 and 42.

### 3.3. Generation of Monoclonal Cell Lines from the BALB-CTA

For a more detailed differentiation between untransformed monolayer cells and transformed, foci-forming cells, a total of 29 monoclonal cell lines were generated from cell foci on Day 35 of the BALB-CTA. These cell lines showed highly heterogeneous growth phenotypes, judged via Giemsa staining (Additional file 1: Supplementary Figure S1). As expected, untransformed control cell lines (WT41–46) grew as a monolayer and showed no basophilic staining. For the MCA/TPA-treated cells, we expected multilayered growth with deep basophilic staining, as was the case in the clones T52, T59, and T69. However, several other morphologies were observed. The clones T56, T57, T58, T63, T64, T65, T67, T68, T70, T72, T73, and T75 showed a mixed population of monolayer and foci-forming cells. Intriguingly, the clones T51, T53, T54, T60, T61, T71, T74, and T77 grew as a monolayer without foci formation or basophilic staining, comparable to control cells. An overview of the cell clones is provided in Table S1.

### 3.4. Metabolic Characterization of Selected Cell Clones

To study the metabolism of these heterogeneous clones in more detail, we chose the control clone WT44, the colony-forming clone T58, and the multilayer-forming clones T52 and T59 (Figure 3A) for further characterization. Metabolic profiling using a Seahorse analyzer revealed wide differences in OCRs resulting from mitochondrial respiration (Figure 3B) and ECARs originating from fermentative/aerobic glycolysis (Figure 3C) among the cell clones. Compared to WT44, the OCR was significantly higher in the clones T52 (256%) and T59 (329%) but similar in T58 (Figure 3B,D). These differences remained during the analysis and after the consecutive injections of the ATP synthase inhibitor oligomycin, the mitochondrial uncoupler FCCP, and the ETC complex III inhibitor antimycin A (Figure 3B). In contrast, the basal ECAR was similar in WT44, T52, and T59 cells but was significantly higher for the clone T58 (147%) (Figure 3C,E). The clone T58 resembled a classic example of Warburg-like metabolic adaptation after cell transformation. The ATP production (Figure 3F) was calculated from the OCR curves for each cell clone (Figure 3B). In comparison to WT44, the ATP production was significantly lower in T58 cells (64%) and significantly higher for the clones T52 (267%) and T59 (348%). Cell growth was determined from elaborated growth curves within one experiment. The duplications per 24 h were comparable for T52 cells (1.2-fold), lower for T58 cells (0.8-fold), and higher for T59 cells (1.9-fold), relative to WT44 (1.2-fold) (Figure 3G). On the basis of these data, different metabolic phenotypes in transformed cells compared with WT44 cells were calculated and defined, with the T52 clone identified as high metabolic, T58 identified as glycolytic, and T59 identified as high metabolic and fast growing (Figure 3H).

### 3.5. Treatment of Cell Clones with Butyrate and Metformin

We recently studied the effects of metformin as a single drug treatment and in combination with standard chemotherapeutic agents in the BALB-TTM [13]. Metformin alone significantly reduced the number of type-III foci whilst increasing the glucose consumption. Since butyrate and metformin act as anti-tumor therapeutics with adverse effects on the glucose consumption of transformed cell foci (Figure 2 and [13]), we aimed to characterize their effects on the energy metabolism of individual cell clones. In line with the heterogeneity of the cell clones, the treatments affected the mitochondrial respiration (Additional File 2: Supplementary Figure S2A,C,E,G) and glycolysis (Additional File 2: Supplementary Figure S2B,D,F,H) differently.

Butyrate had no effect on the basal OCR of WT44, T52, and T58 cells (Figure 4A–C), but increased basal OCR values significantly in the T59 clone (150%) (Figure 4D). In contrast, metformin significantly reduced the basal OCR in WT44 (59%), T52 (43%), and T58 cells (46%), but not in the T59 clone (83%) (Figure 4A–D). Basal ECAR values were reduced in WT44 cells after treatment with butyrate (73%) and increased with metformin (126%) (Figure 4E). Butyrate similarly reduced the basal ECAR in T52 (72%) (Figure 4F) and T59 cells (78%) (Figure 4H) and led to a partial reversion of the Warburg effect in the T58 clone (77%) (Figure 4G). Metformin did not affect the glycolysis of the clones T52 and T58 but increased it significantly in T59 cells (167%) (Figure 4F–H). Again, the ATP production was determined from OCR curves (Additional File 2: Supplementary Figure S2A,C,E,G). It was significantly reduced after treatment with metformin in all four tested clones (Figure 4I–L). Interestingly, butyrate specifically enhanced the ATP production of the T59 clone (Figure 4L). In addition, we observed that butyrate reduced the cell growth of WT44 (64%) (Figure 4M), T52 (62%) (Figure 4N), and T59 cell clones (51%) (Figure 4P). However, no significant effect of the butyrate treatment was observed in T58 cells (78% reduction) (Figure 4O). Metformin slightly, although not significantly, increased the growth of the transformed T52, T58, and T59 cell clones, but had no effect on the growth of WT44 cells (Figure 4M–P). The cell viability for all conditions and among cell clones was always >90% (not shown).

**Figure 5:**
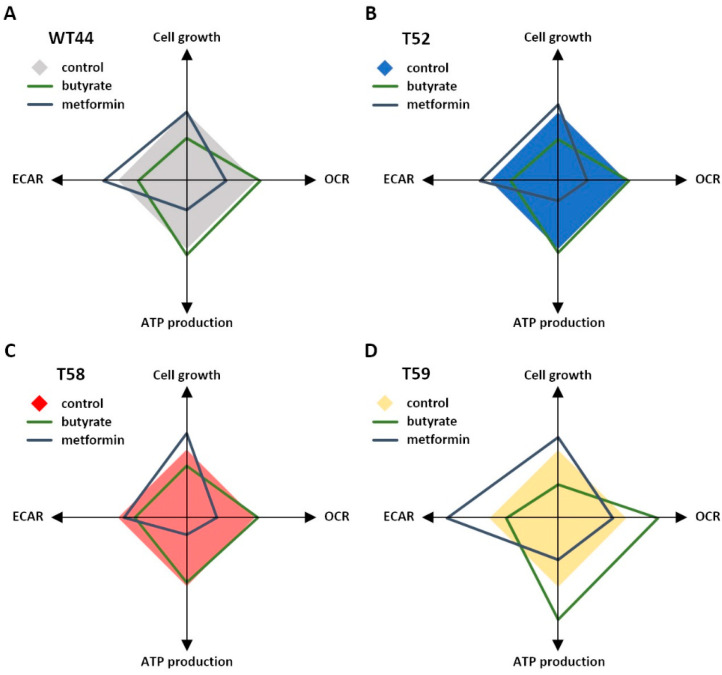
Illustration of the metabolic changes. The effects of butyrate or metformin treatment on cell growth, basal OCR, ATP production, and basal ECAR are illustrated relative to untreated cells for the clones (**A**) WT44, (**B**) T52, (**C**) T58, and (**D**) T59.

The effects of the butyrate and metformin treatments on the individual cell clones are summarized and illustrated in Figure 5. Although metformin and butyrate induced adverse metabolic alterations, the individual effects of these agents were comparable in all tested cell clones, despite their strong metabolic heterogeneity. The high-metabolic and fast-growing clone T59 showed the strongest effect for both agents on its energy metabolism, whereas butyrate promoted a shift into mitochondrial respiration in the glycolytic clone T58.

## 4. Discussion

In the present study, we isolated 29 monoclonal cell lines with different growth behavior from malignant type-III cell foci of the BALB-CTA and untransformed control cells. In a subset of these clones, we studied their metabolic phenotypes and analyzed alterations in energy metabolism after treatment with butyrate and metformin. In a previous study, we established the BALB-TTM with commonly used chemotherapeutic agents in combination with metformin as a new model to investigate therapy effectiveness [13]. We observed that metformin as a single agent increased the glucose consumption within the BALB-TTM in non-toxic concentrations whilst showing anti-carcinogenic effects. In our current study, we observed anti-carcinogenic effects of butyrate alongside metformin in the BALB-TTM, despite their opposing effects on cell metabolism, making the BALB-TTM and derived monoclonal cell lines a suitable system to mimic and study the response of a heterogeneous tumor cell population to novel potential therapeutic agents.

Tumor heterogeneity is a crucial factor for cancer progression, resistance to therapy, and recurrence [30]. Congruently, specific tumor genotypes have led to personalized targeted therapies, which have shown better outcomes than generalized approaches [31,32,33]. Established cancer cell lines offer a potent tool for basic cancer research and are fundamental for the development of therapies [34]. However, these cell lines often represent only a subpopulation of a tumor from a specific tissue of origin and cannot simulate the intra-tumor and inter-individual heterogeneity of the transformed cells [35]. Three-dimensional in vitro cell culture models and co-culture models try to mimic the biological situation of tumors, as well as the intra-tumor heterogeneity, but due to their complexity, the usage of these models is limited [36].

The non-malignant mouse fibroblasts used for the BALB-CTA simulate the process of malignant cell transformation in vitro. After the application of MCA/TPA, several morphologically different heterogeneous foci consisting of transformed cells arise, showing different growth behaviors and activation of molecular signaling pathways [37]. Moreover, cells from the edge of these foci differ from the inner cell mass [12], which is similar to the situation in solid tumors. When injected into mice, foci-forming cells are able to induce tumors [38,39], underlining their malignant status. Therefore, the BALB-CTA and derived cell lines offer a simplified unbiased tool for mechanistic cancer research that considers inter- and intra-tumor heterogeneities. Although transformed fibroblasts do not represent a specific type of cancer, they show several characteristics that play an important role in cancer development [40]. Butyrate, as a product of bacterial fermentation of dietary fibers in the intestine, is the main energy source of colonocytes and is essential for the maintenance of colonic homeostasis [41]. Moreover, butyrate serves as an anti-carcinogenic drug with potential in anti-tumor therapy [42,43]. Depending on the diet and the composition of the microbiota, concentrations of 11 to 25 mM butyrate were measured in human feces [41]. In the colon of mice, different butyrate concentrations were detected ranging from 3.5 mM in the proximal segments to 0.5 mM in the distal segments [14]. Hence, the applied concentrations of 0.1 and 1 mM in this study are physiologically relevant for general mechanistic investigations.

Butyrate at 1 mM showed anti-proliferative effects on the non-transformed BALB/c fibroblasts and a foci-reducing effect in the BALB-TTM. Since we previously observed that an inhibition of cell proliferation in non-transformed cells does not necessarily lead to a decrease in foci formation [12], we proved here the anti-carcinogenic therapeutic effects of butyrate using the BALB-TTM. Butyrate is able to reduce glucose consumption in cancer cells [44], and an inhibition of glucose metabolism shows anti-carcinogenic effects [45]. Therefore, we assume an inhibition of glycolysis in the BALB-TTM as the underlying mechanistic cause of its anti-cancer effects against MCA/TPA-treated, foci-forming cells. So far, we have demonstrated here, together with our previous study, that both metformin [13] and butyrate show tumor therapeutic effects in the BALB-TTM, although both substances affect glucose metabolism in adverse ways.

For a more specific investigation and better differentiation between the untransformed monolayer cells and the transformed foci-forming cells, we isolated 6 untransformed and 23 transformed monoclonal cell lines that originated from different cell foci. The growth behavior and Giemsa staining were highly heterogeneous among the malignant cell clones. All untransformed clones had a similar fibroblast-like morphology growing as a monolayer and being Giemsa negative. Among the transformed cell lines, only three showed the expected multilayered cell growth resulting in deep basophilic staining. The reasons for this unexpected finding are unknown and must be further investigated.

Interestingly, our selection of cell clones showed markedly different results concerning cell growth, ATP production, oxidative metabolism, and glycolysis, confirming that the MCA/TPA treatment results in a heterogeneous population of transformed cells. Although the growth curves were performed as a single experiment, this elaborate method offers robust results even without a statistical analysis. We determined four different phenotypes, and defined them as control (WT44), high metabolic (T52), glycolytic (T58), and high metabolic and fast growing (T59). This observation is similar to the in vivo situation where tumors show different metabolic phenotypes [46,47]. Intriguingly, the high-metabolic and fast-growing cell clone T59, resembling an aggressive or hyper-mutated tumor entity, was highly affected by butyrate and metformin treatments. Moreover, a metabolic shift from aerobic glycolysis into mitochondrial respiration and energy production was observed in the T58 clone, which showed a strong Warburg-like metabolic phenotype before. With the generation of the monoclonal cell lines, we could now, for the very first time, investigate specific effects of butyrate and metformin on transformed cells with different metabolic phenotypes and compare them with the untransformed control cells from which they all originate. This may help in future to predict and optimize the outcome of anti-tumor therapies targeting the energy metabolism of malignant cells.

To a limited extent, the SCFA butyrate is metabolized and can act as an energy source before it accumulates in the cell nucleus and alters gene expression as an inhibitor of histone deacetylases [14]. In addition, it was shown that butyrate could induce a metabolic switch in several cancer cells, which results in less glycolytic and more oxidative metabolism [28,29]. We confirmed this in our cell clones. In WT44, T52, and T58 cells, butyrate inhibited cell growth, associated with an inhibition of glycolysis, without affecting the oxidative metabolism. Interestingly, in the high-metabolic and fast-growing cell clone T59, butyrate induced a metabolic shift. Oxidative metabolism and ATP production were increased, while glycolysis was inhibited. This metabolic change connects to a strong reduction in cell growth without inducing apoptosis. Importantly, butyrate inhibited glycolysis in all of the four clones to the same extent of about 75% compared to untreated values, independently of the basal glycolytic level.

Metformin is a potent inhibitor of the mitochondrial ETC complex I, leading to disturbed oxidative metabolism [48] and an increase in glucose consumption in peripheral tissues like muscle and kidney [15,16]. Interestingly, an adverse effect is described in some cancer cell lines where metformin is able to reduce glucose consumption [17,18,19,20,21]. Congruently, in the untransformed cell clone WT44, metformin potently inhibited oxidative metabolism, resulting in an increased glycolytic rate and, finally, lower ATP levels. For the transformed high-metabolic and glycolytic cell clones T52 and T58, metformin disturbed oxidative metabolism, leading to lower ATP levels without affecting glycolysis. The high-metabolic and fast-growing cell clone T59 is again of special interest, because metformin significantly increased glycolysis but could not impair oxidative metabolism. Nevertheless, a decrease in ATP production was observed. The molecular background remains elusive and must be further addressed in a following study. An adaption of the glucose concentration in the medium would be of great interest. Cells with defects in glucose utilization or mitochondrial function are highly sensitive to low-glucose conditions and to biguanide treatment [49]. Considering these facts, the effects of metformin under low-glucose conditions on the cell clone T58, which was not able to adapt glycolysis after biguanide treatment, should be addressed in a following study. In addition, it was shown that distinct metabolic phenotypes show different responses to metformin treatment under varying glucose conditions [50]. The generated cell clones offer the great possibility to study the dependency of metformin on the glucose availability and on different metabolic phenotypes.

Although therapeutic effects were observed for metformin in the BALB-TTM [13], the cell growth remained unaltered during 48 h of treatment in all four cell clones tested. It is plausible that the observed metabolic shift could lead to decreased foci formation in the BALB-TTM after a longer treatment period of 10 days.

Taking into account that metabolic profiling is becoming more and more important for targeted anti-tumor therapies [51], the isolated cell clones offer new insights into phenotypic effects and could offer a potent tool to study the effectiveness of anti-cancer drugs in vitro. Notably, the high-metabolic cell clone T52 showed significantly higher oxidative metabolism and ATP production compared to the control clone WT44, but butyrate and metformin affected energy metabolism in both clones equally. In contrast, the metabolism of cell clone T59 was more comparable to that of clone T52 but reacted highly differently and more sensitively to the butyrate and metformin treatment. These results suggest that butyrate and metformin affect energy metabolism independently of the metabolic phenotype. Remarkably, butyrate and metformin showed anti-carcinogenic effects in the BALB-TTM despite the observed heterogeneity of the transformed cells with distinct metabolic phenotypes, indicating that tumor cells are, in general, highly sensitive to changes in their energy metabolism.

## 5. Conclusions

In summary, we were able to establish several transformed cell clones that show highly heterogeneous metabolic and morphologic phenotypes. The individual metabolism of each cancer cell seems to be highly relevant in terms of responsiveness to the applied drugs. As we showed for butyrate and metformin, cell clones with comparable metabolic profiles reacted differently, while cell clones with distinct phenotypes showed a comparable metabolic shift. Butyrate and metformin showed anti-carcinogenic effects in the BALB-TTM. Interestingly, these agents affected the glucose consumption in an adverse way. While butyrate was able to reduce it, metformin increased glycolysis. The isolated cell clones enable direct comparison of untransformed and malignant transformed cells from the same origin and offer a strong tool for further mechanistic studies.

## Data Availability

The datasets used and/or analyzed during the current study are available from the corresponding author on reasonable request.

## Supplementary Materials

The following are available online at https://www.mdpi.com/article/10.3390/biom11121831/s1, Figure S1: Giemsa-stained cell clones, Figure S2: Seahorse results after treatment of cell clones with butyrate and metformin, Table S1: Overview of the cell clones.
