# Supplementary material for: Butyrate and Metformin Affect Energy Metabolism Independently of the Metabolic Phenotype in the Tumor Therapy Model
Source: Biomolecules. 2021 Dec 4;11(12):1831. doi: 10.3390/biom11121831 (PMC8699353; doi:10.3390/biom11121831)
Supplement: Supplementary file 1 [file biomolecules-11-01831-s001.zip › biomolecules-1446958-supplementary.pdf]

Figure S1: Giemsa stained cell clones. The isolated cell clones were cultivated for 16 days, fixed with methanol and stained with Giemsa solution.

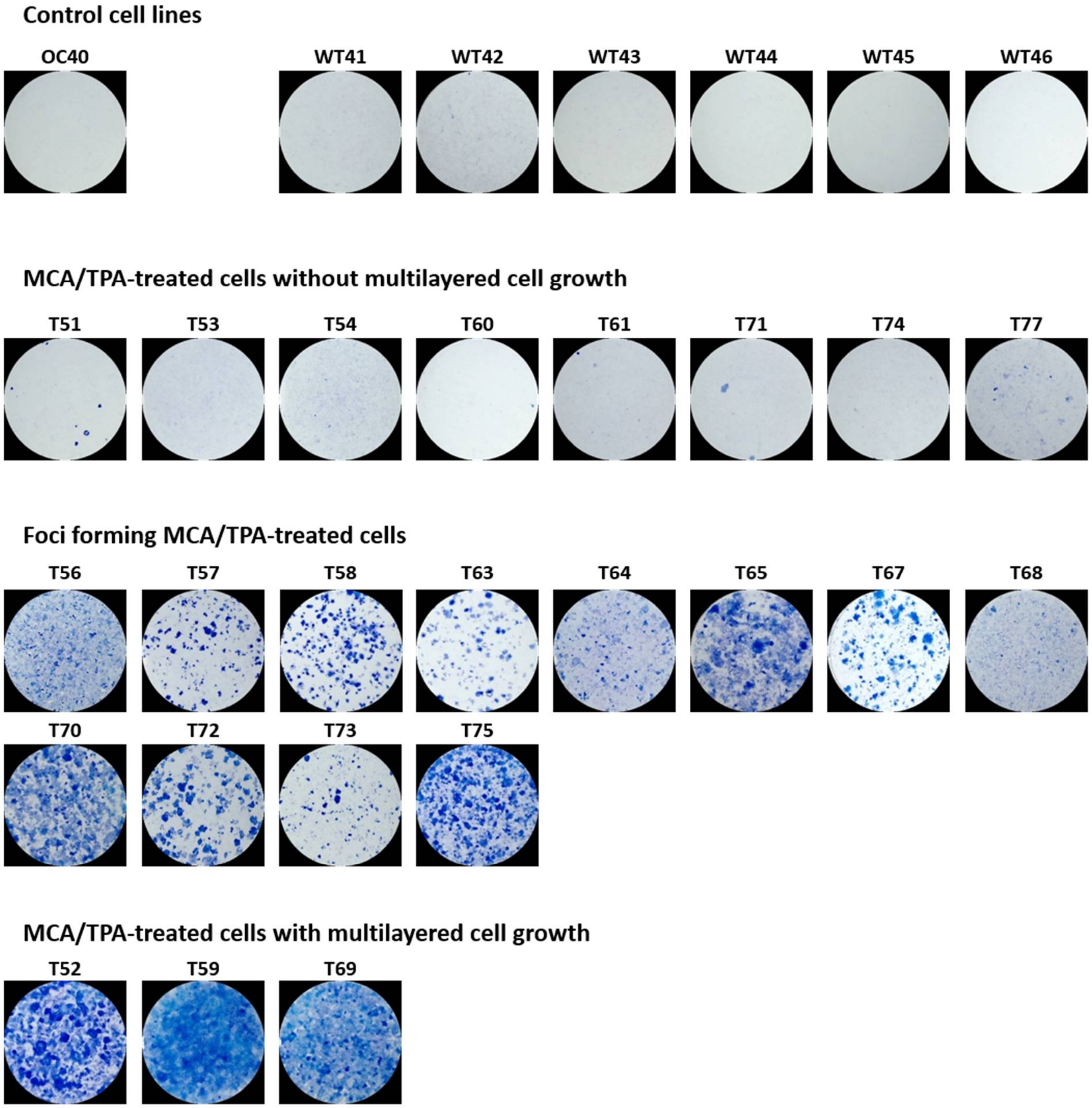

Figure S2. Seahorse results after treatment of cell clones with butyrate and metformin**.** Cell clones were seeded into 24 well Agilent Seahorse XF Cell Culture Microplates, treated with 1 mM butyrate or 1 mM metformin for 24 h following a Seahorse XF Cell Mito Stress assay. The oxygen consumption rates (OCR) are shown relative to untreated control cells for (A) WT44, (C) T52, (E) T58 and (G) T59 cells. The extracellular acidification rates (ECAR) are shown relative to untreated control cells for the clones (B) WT44, (D) T52, (F) T58 and (H) T59. Data are given as mean ± SEM of three to four biological replicates.

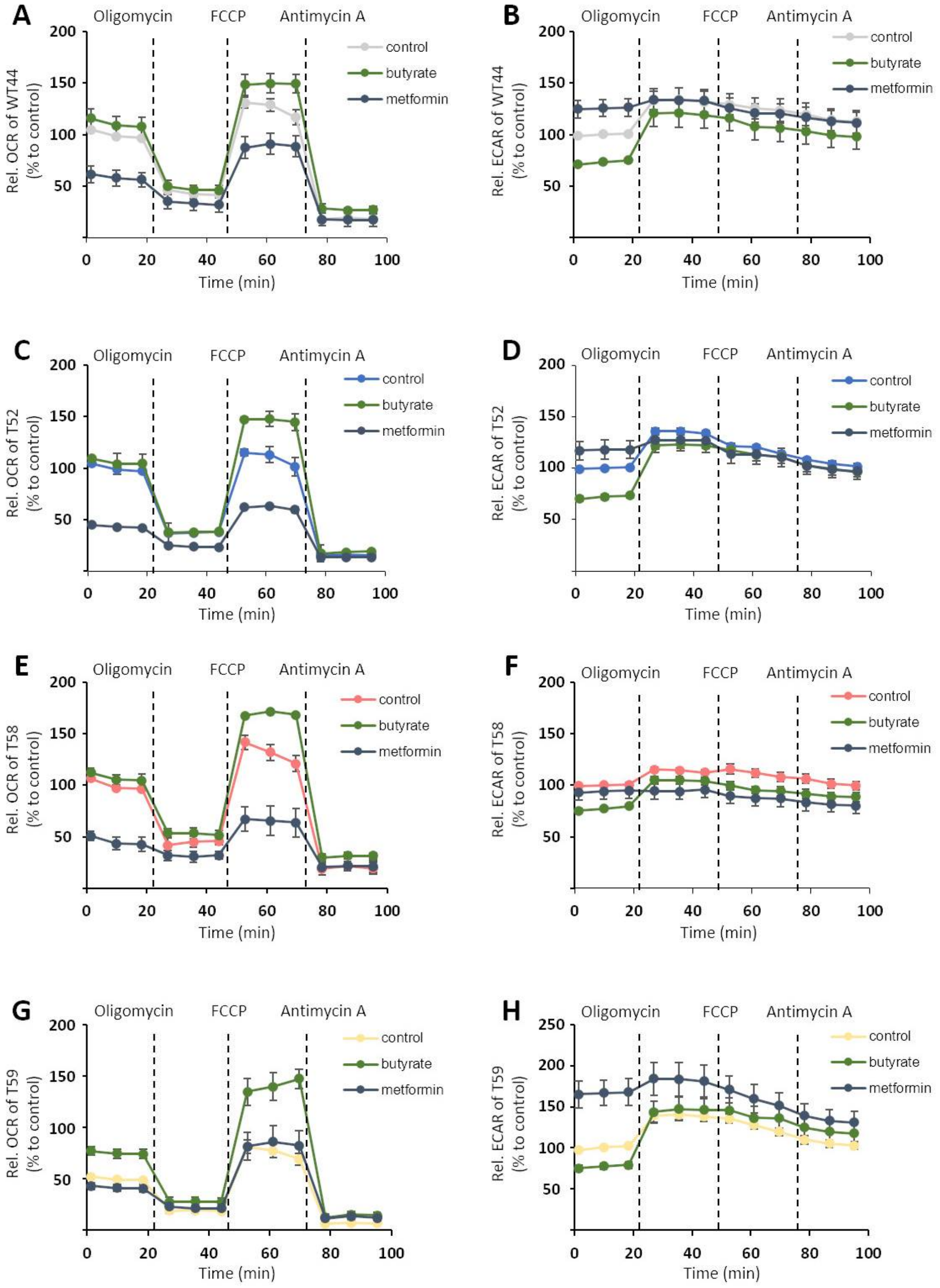

Table S1: Overview of the cell clones.

Monoclonal cell line	Doubling time [h]	Growth characteristics
OC40	17,1	Monolayer, post-confluent growth inhibition
WT41	20,5	Monolayer, post-confluent growth inhibition
WT42	22,8	Monolayer, post-confluent growth inhibition
WT43	27,0	Monolayer, post-confluent growth inhibition
WT44	20,3	Monolayer, post-confluent growth inhibition
WT45	19,8	Monolayer, post-confluent growth inhibition
WT46	18,3	Monolayer, post-confluent growth inhibition
T51	20,9	Monolayer, post-confluent growth inhibition
T52	19,5	Multilayer, highly invasive
T53	17,9	Monolayer, post-confluent growth inhibition
T54	18,9	Monolayer, post-confluent growth inhibition
T56	14,3	Small foci forming, clonal subpopulations
T57	15,4	Foci forming, clonal subpopulations
T58	29,2	Foci forming, clonal subpopulations
T59	12,9	Multilayer, highly invasive
T60	18,7	Monolayer, post-confluent growth inhibition
T61	14,5	Monolayer, post-confluent growth inhibition
T63	20,0	Foci forming, clonal subpopulations
T64	18,5	Small foci forming, clonal subpopulations
T65	17,1	Foci forming, clonal subpopulations
T67	17,9	Foci forming, clonal subpopulations
T68	19,7	Small foci forming, clonal subpopulations
T69	13,3	Multilayer, highly invasive
T70	13,2	Foci forming, clonal subpopulations
T71	17,4	Monolayer, post-confluent growth inhibition
T72	22,9	Foci forming, clonal subpopulations
T73	21,5	Foci forming, clonal subpopulations
T74	15,9	Monolayer, post-confluent growth inhibition
T75	17,9	Foci forming, clonal subpopulations
T77	22,8	Monolayer, post-confluent growth inhibition
